# The impact of smoking on recurrence and progression of non-muscle invasive bladder cancer: a systematic review and meta-analysis

**DOI:** 10.1007/s00432-022-04464-6

**Published:** 2022-11-21

**Authors:** Aleksander Ślusarczyk, Piotr Zapała, Łukasz Zapała, Piotr Radziszewski

**Affiliations:** Department of General, Oncological and Functional Urology, Medical University of Warsaw, Lindleya 4, 02-005 Warsaw, Poland

**Keywords:** Non-muscle-invasive bladder cancer, Smoking, Current smoker, Recurrence, Progression

## Abstract

**Objectives:**

Although smoking is a well-recognized causative factor of urothelial bladder cancer and accounts for 50% of cases, less is known about the prognostic significance of smoking on non-muscle invasive bladder cancer (NMIBC) prognosis. This systematic review and meta-analysis aimed to evaluate the effect of smoking on the risk of NMIBC recurrence and progression.

**Materials and methods:**

We systematically searched Medline, Web of Science and Scopus databases for original articles published before October 2021 regarding the effect of smoking on NMIBC recurrence and progression. Information about smoking status and the number of events or odds ratio or hazard ratio for event-free survival must have been reported to include the study in the analysis. Quality In Prognosis Studies tool was utilized for the risk of bias assessment.

**Results:**

We selected 64 eligible studies, including 28 617 patients with NMIBC with available data on smoking status. In a meta-analysis of 28 studies with 7885 patients, we found that smokers (current/former) were at higher risk for recurrence (OR = 1.68; 95% CI 1.34–2.09; *P* < 0.0001) compared to never smokers. Subgroup analysis of 2967 patients revealed that current smokers were at a 1.24 higher risk of recurrence (OR = 1.24; 95% CI 1.02–1.50; *P* = 0.03) compared to former smokers. A meta-analysis of the hazard ratio revealed that smokers are at higher risk of recurrence (HR = 1.31; 95%CI 1.15–1.48; *P* < 0.0001) and progression (HR = 1.18; 95%CI 1.08–1.29; *P* < 0.001) compared to never smokers. Detrimental prognostic effect of smoking on progression, but not for recurrence risk was also noted in the subgroup analysis of high-risk patients (HR = 1.30; 95%CI 1.09–1.55; *P* = 0.004) and BCG-treated ones (HR = 1.15; 95%CI 1.06–1.25; *P* < 0.001).

**Conclusion:**

In conclusion, patients with non-muscle invasive bladder cancer and a history of smoking have a worse prognosis regarding recurrence-free and progression-free survival compared to non-smokers.

**Supplementary Information:**

The online version contains supplementary material available at 10.1007/s00432-022-04464-6.

## Introduction

Urothelial bladder cancer (UBC) is ranked as the seventh most common malignancy worldwide in men and seventeenth in women. Smoking is a well-evidenced, strong causative factor responsible for the development of approximately 50% of bladder tumours (Burger et al. 2013). Cumulative exposure to tobacco smoke is associated with a strong risk of cancer development in the urinary tract (Zeegers et al. 2000; Brennan et al. 2000). Smoking cessation could mitigate the risk of bladder cancer development (Zeegers et al. 2000). On the other hand, one of the prospective surveys showed that only 36% of patients who presented to a urology clinic were aware of bladder cancer risk caused by smoking compared to 98% aware of the link between smoking and lung cancer (Nieder et al. 2006). Despite the detrimental effect of smoke on health including cardiovascular and pulmonary functions and the risk of other cancer development, approximately 30% of patients remain active smokers at UBC diagnosis (Grotenhuis et al. 2014). Strikingly a prospective observational study showed that only 34.5% of smokers have quit smoking after the diagnosis (Serretta et al. 2020).

Available meta-analysis of 17 studies on 13 777 patients indicates that response to neoadjuvant chemotherapy (NAC) and outcomes of radical cystectomy (RC) for muscle-invasive bladder cancer (MIBC) are compromised in smokers (Cacciamani et al. 2020 Oct). To date studies assessing the impact of smoke on non-muscle invasive bladder cancer (NMIBC) prognosis are not convincing, with several retrospective analyses showing conflicting results (Grotenhuis et al. 2014; Ogihara et al. 2016; D’Andrea et al. 2017; Ślusarczyk et al. 2019). Due to the following reasons, smoking status has not been included in the most widely used risk tables for NMIBC recurrence and progression (Fernandez-Gomez et al. 2009; Sylvester et al. 2006). Simultaneously, limited evidence impairs consecutive smoking cessation counselling in UBC patients and tailoring the treatment protocol in smokers. One meta-analysis including 11 studies on the cohort of 7210 patients showed a worse recurrence-free survival (HR = 1.27; 95% CI 1.09–1.46) in current compared to never smokers diagnosed with NMIBC (Osch et al. 2016). Due to the limited number of patients included in the risk assessment of PFS according to the smoking status, the meta-analysis failed to show a statistically significant association(Osch et al. 2016).

The aim of this systematic review and meta-analysis was to evaluate the effect of smoking on the risk of NMIBC recurrence and progression.

## Materials and methods

### Study selection

A systemic literature review was performed in accordance with the Preferred Reporting Items for Systematic Reviews and Meta-analyses statement (PRISMA guideline, for details please see the Supplementary file) to identify studies published before October 2021. We searched Medline (Pubmed), Scopus and Web of Science databases using the following terms: “((progression OR progression-free survival OR muscle-invasive) AND (non-muscle invasive bladder cancer OR NMIBC) AND (Risk factors OR Smoking OR smoke OR cigarette OR tobacco)) OR ((progression OR recurrence-free survival OR muscle-invasive OR recurrence) AND (non-muscle invasive bladder cancer OR NMIBC) AND (Smoking OR smoke OR cigarette OR tobacco))” and “(recurrence OR progression OR survival) AND (non-muscle invasive bladder cancer OR NMIBC) AND (Smoking OR smoke OR cigarette OR tobacco)”, respectively.

**Fig. 1:**
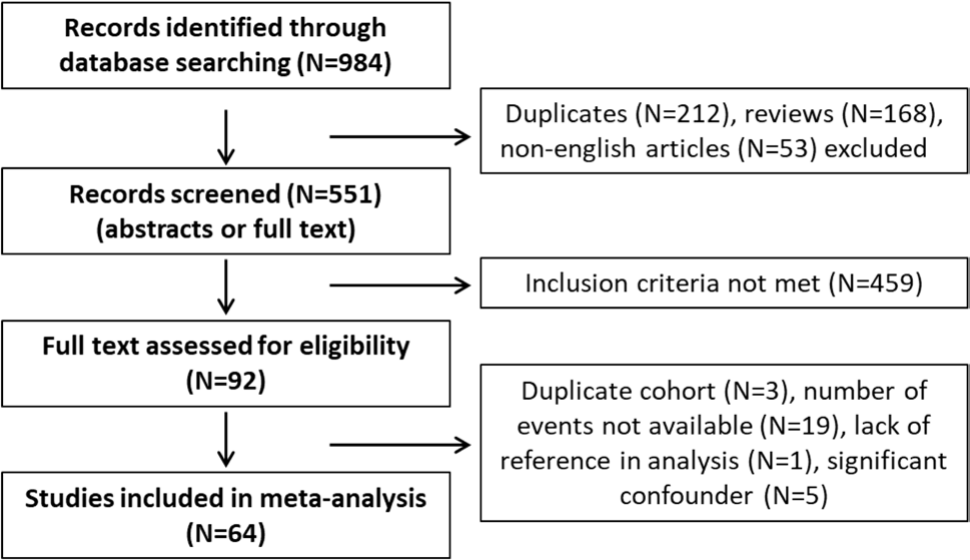
Flowchart for study selection process using Medline, Web of Science and Scopus databases to identify the original articles demonstrating the effect of smoking on prognosis in non-muscle-invasive bladder cancer

Relevant citations of identified papers were manually searched to retrieve any further studies not found using algorithmic queries. Studies were included only if the information regarding smoking status (current, former, never smoker OR smoker vs non-smoker) and NMIBC recurrence and/or progression risk were available(for details see the selection flowchart Fig. 1).

The inclusion criteria were as follows: provided data on smoking status (smoker/nonsmoker OR current/former/never smoker) and its association with the risk of recurrence and/or progression in non-muscle invasive bladder cancer. Either the number of events, event rate or odds ratio or hazard ratio for event-free survival must have been reported to include the study in the analysis. Studies in which the odds ratio for recurrence/progression according to the smoking status were available but the number of events were not reported and could not be recalculated were excluded (e.g., (Rausch et al. 2014; Pastore et al. 2015; Nerli et al. 2018; Holz et al. 2017)). In all studies the definition of the progression included the development of muscle-invasive disease (MIBC) and in the majority of studies any increase in T stage or grade was also regarded as progression. We excluded the studies reporting only smoking intensity or smoking time but not precisely the smoking status (former/current/never or smoker/nonsmoker) (Andrade et al. 2020). Studies with a single analysis for the cohort of both NMIBC and MIBC or studies in which the information on tobacco use was not precise were also excluded (Nerli et al. 2018; Zhang et al. 2021; Ahirwar et al. 2008).

Primary endpoints were the risk of tumour recurrence and/or progression. Abstracts were screened for eligibility and selected original papers were studied as full text. The data was retracted from prospective studies and retrospective studies including accepted manuscripts and their supplementary data files. Two independent investigators performed the screening and extracted data. In the event of duplicated analyzes of same cohorts in different publications, the study with more robust data was included.

### Risk of bias assessment

Studies differently reported smoking status, which is the unavoidable bias of our meta-analysis. The reported smoking history is presented for each study in Table 1. Doubts regarding the smoking status interpretation occurred due to a lack of definitions and unclear reports in several studies. Several ways of reporting the smoking status were provided in different papers as follows: smokers vs non-smokers, smoking (yes/no) and smoking status (yes/no), and definitions were most commonly not available. History of smoking (yes/no) was also a common way of reporting and was interpreted as ever smoking vs never smoking (Shen et al. 2016; Yang et al. 2021; Mano et al. 2015). Some studies provided an explanation of the smoking status (yes vs no) (Kim et al. 2018; Ferro et al. 2020) as ever smoking (former/current) vs never smoking (Kim et al. 2018). Similarly, the variable “smoking (yes vs no)” and “smokers vs non-smokers” (Kang et al. 2014; Gangawar et al. 2010) was interpreted as a comparison between ever-smoking vs never-smoking but explained only in a few studies as cited. Only 30 studies provided an analysis of outcomes (hazard ratio or number or rate of recurrence/progression) according to detailed smoking status (never/former/current smoking). To assess the robustness of performed analysis, sensitivity analyses were performed including studies with the most reliable data on smoking status and the risk of recurrence and progression (pooled hazard ratio).

**Fig. 2:**
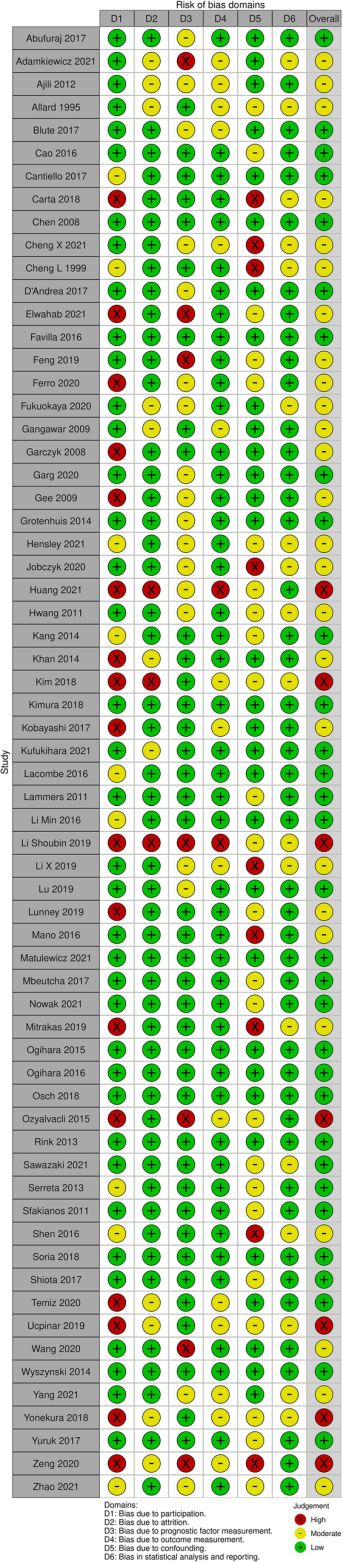
Risk of bias assessment using Quality In Prognosis Studies tool (QUIPS)

The quality of studies and the risk of bias were assessed using the Quality In Prognosis Studies tool (QUIPS). In the bias evaluation with QUIPS, the following six domains were analyzed: study participation (sampling bias), study attrition (attrition bias), prognostic factor measurement, outcome measurement (ascertainment bias), study confounding, and statistical analysis and reporting (Hayden et al. 2013). The overall risk of bias was assessed for each study. Robvis tool was used to visualize the risk of bias in all selected studies. Thirty-three out of sixty-four studies were characterized by a moderate to high-risk of bias (see Fig. 2 for details).

### Statistical analysis

Groups of current smokers vs former smokers and former vs never smokers were compared in terms of NMIBC recurrence risk (odds ratio). Recurrence-free survival was compared between current vs never smokers and former vs never smokers (pooled hazard ratio). Groups of smokers (current or former) vs non-smokers (never smokers) were compared in terms of recurrence and progression risk (odds ratio and pooled hazard ratio). Odds ratios supplemented with 95% confidence intervals were generated based on contiguous tables containing raw data with the number of events in smokers/non-smokers or current/former/never smokers. Hazard ratios for time to recurrence or progression were retrieved from studies with respective 95% confidence intervals and the pooled hazard ratio was calculated. Adjusted hazard ratios were preferably extracted whenever available. We summarized the data using a random-effects model. Forest plots with 95% confidence intervals were generated. To assess the heterogeneity we used the Chi-square test for the degrees of freedom evaluation and the *I*^2^ statistic was calculated. *I*^2^ values of 25, 50, and 75% were regarded as a border of small, moderate, and large amounts of heterogeneity. All data were introduced and analyzed in the Review Manager (RevMan) version 5.4. software.

## Results

**Fig. 3:**
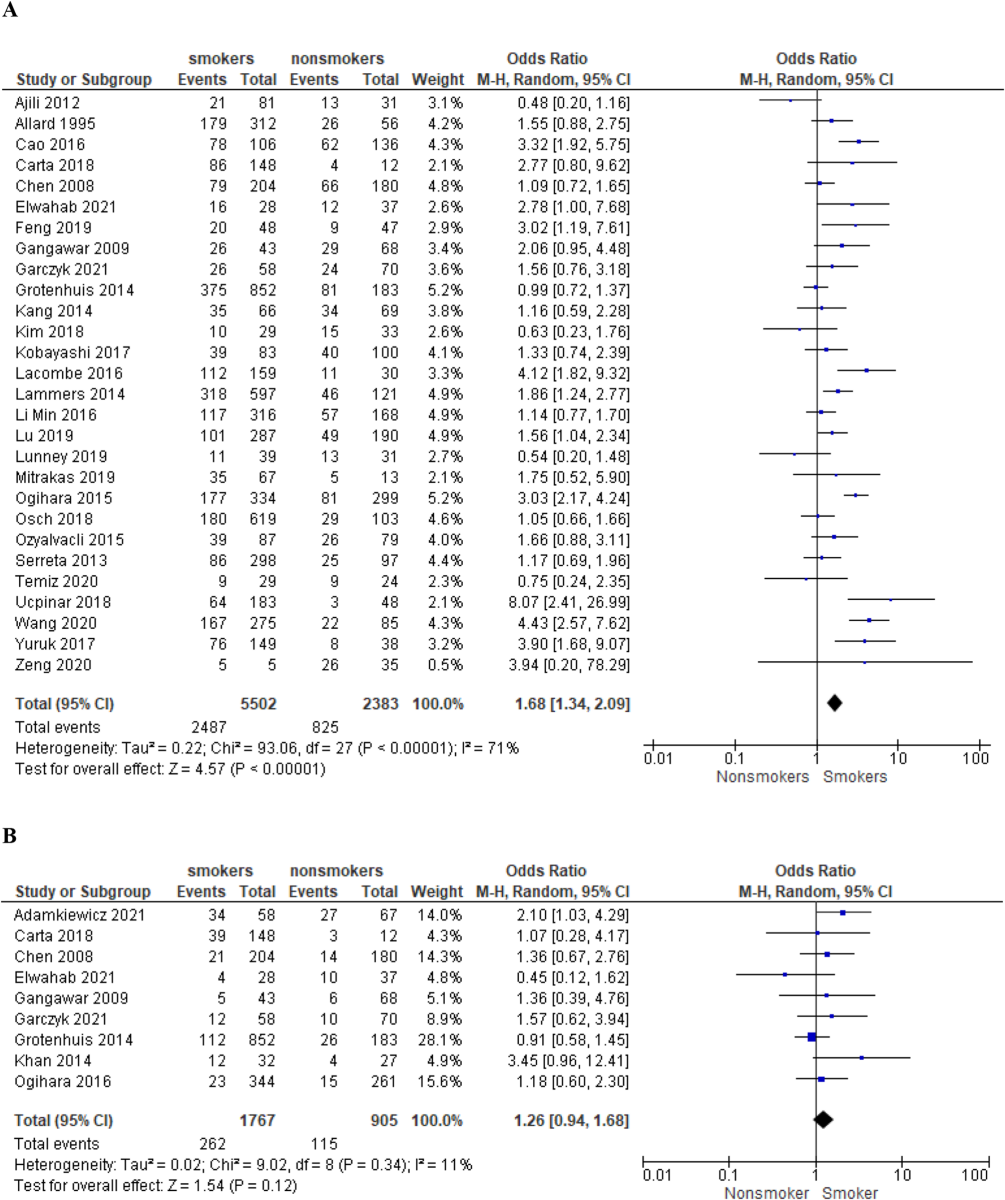
The association of smoking with recurrence risk (**A**) and progression risk (**B**) in patients with non-muscle invasive bladder cancer

**Fig. 4:**
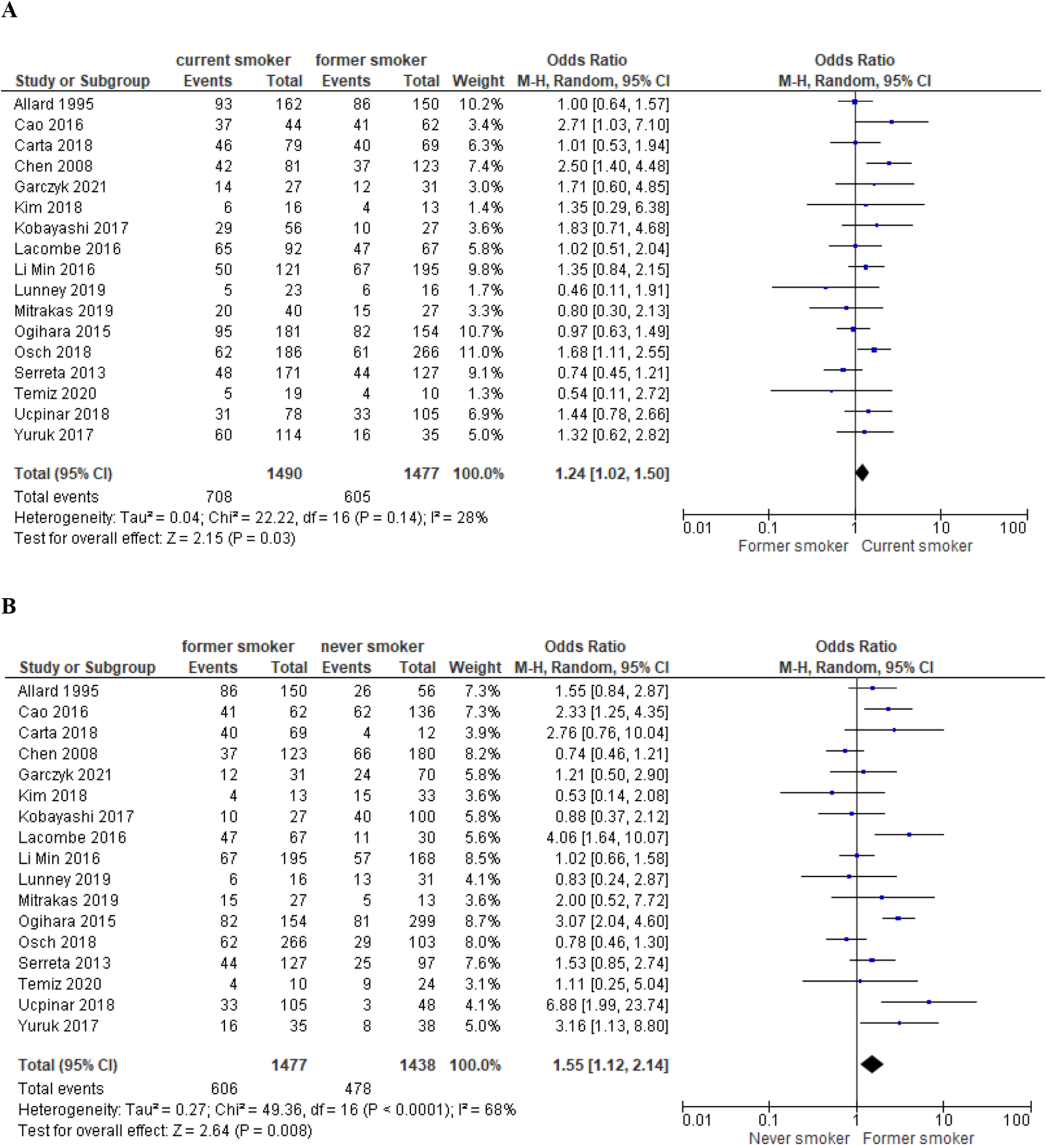
Recurrence risk in current compared to former smokers (**A**) and in former compared to never smokers (**B**) with non-muscle invasive bladder cancer

**Fig. 5:**
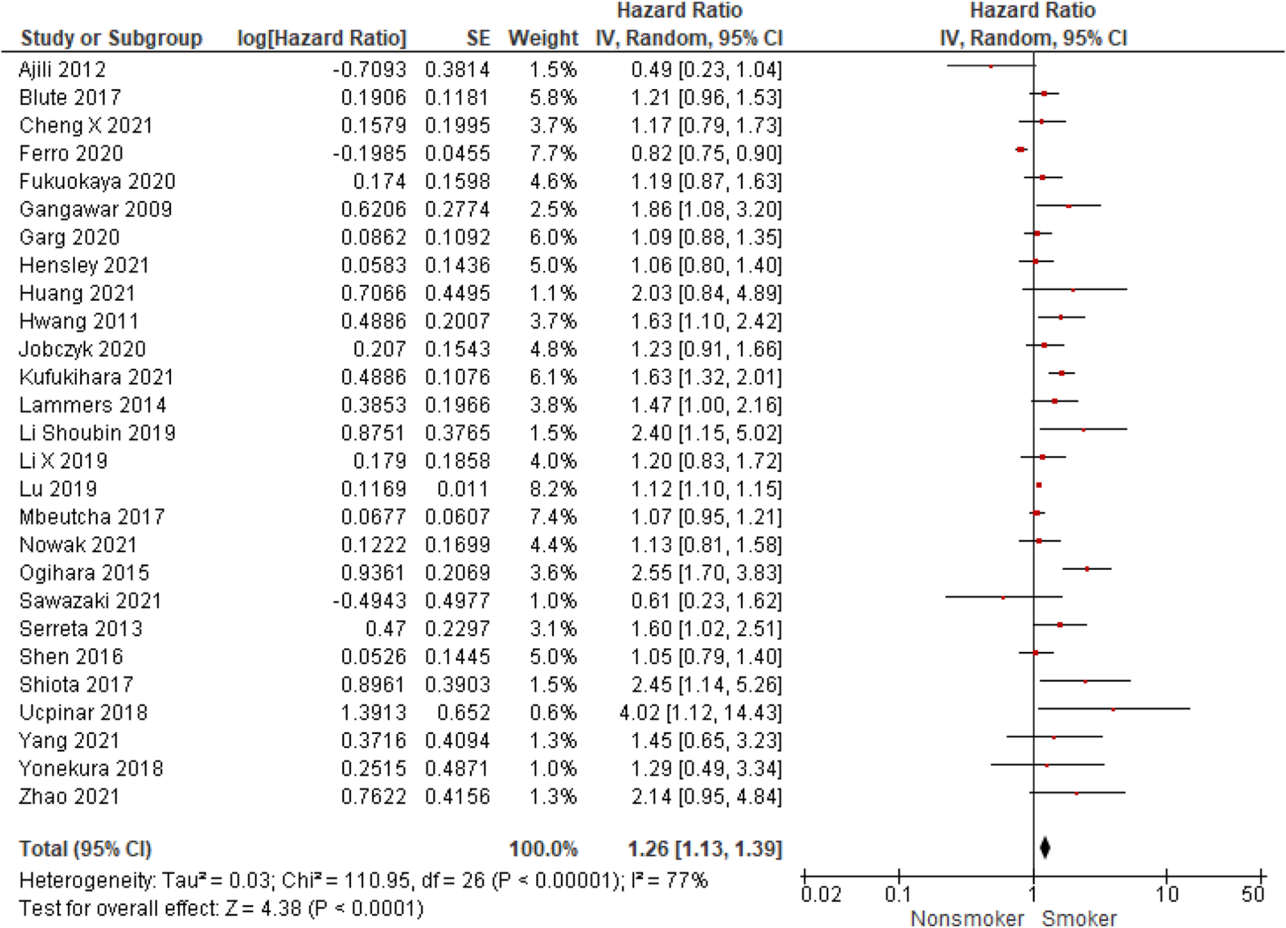
The association of smoking with recurrence-free survival in patients with non-muscle invasive bladder cancer

**Fig. 6:**
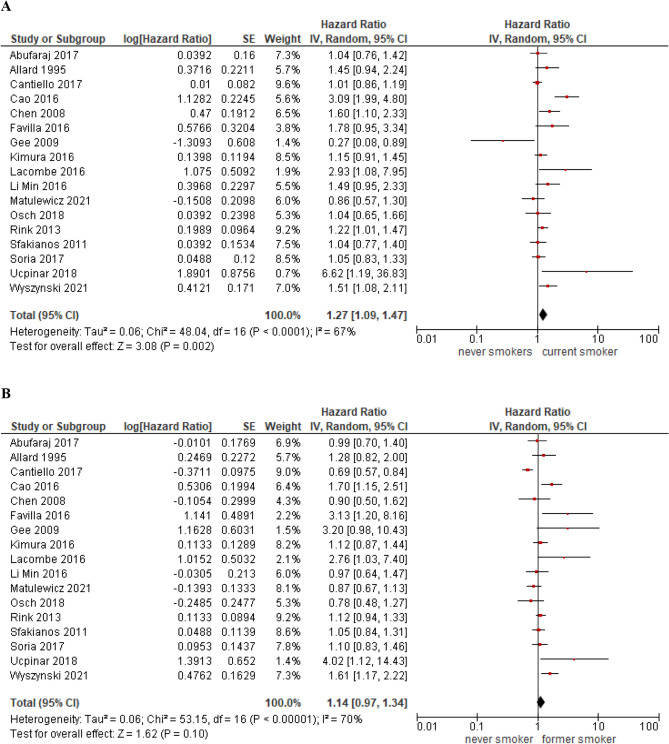
Recurrence-free survival in current compared to never smokers (**A**) and in former compared to never smokers (**B**) with non-muscle invasive bladder cancer

**Fig. 7:**
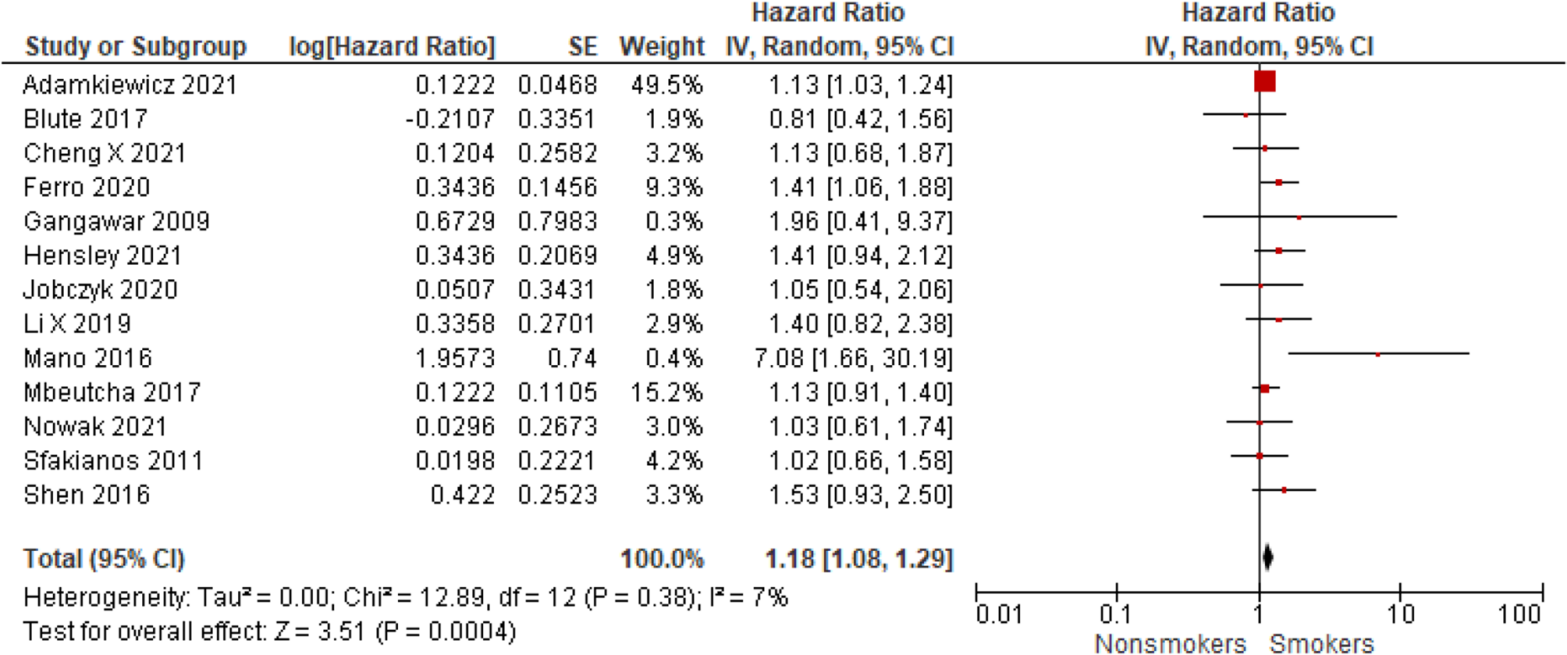
The association of smoking with progression-free survival in patients with non-muscle invasive bladder cancer

**Fig. 8:**
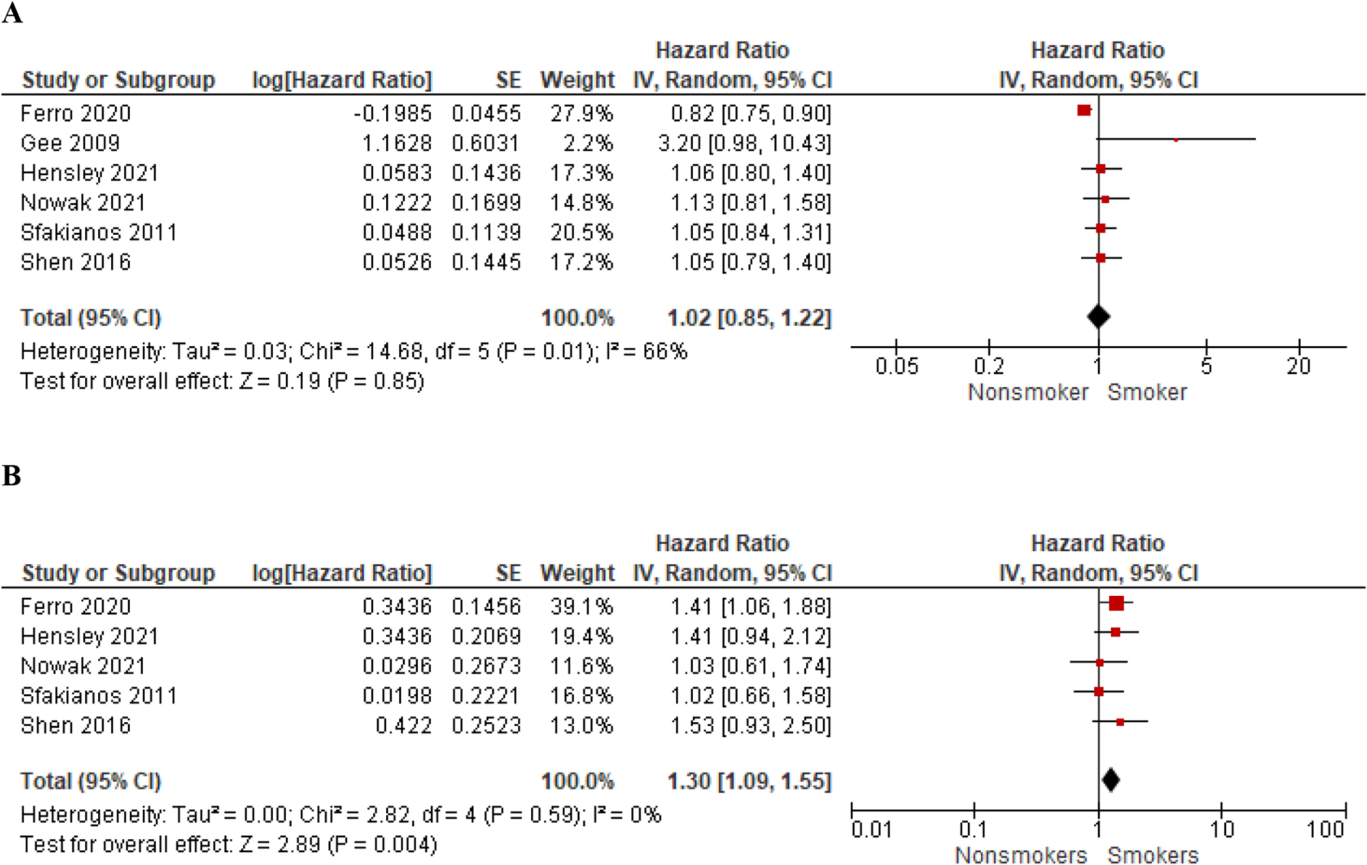
The association of smoking with recurrence-free survival (**A**) and progression-free survival (**B**) in high-risk non-muscle invasive bladder cancer

**Fig. 9:**
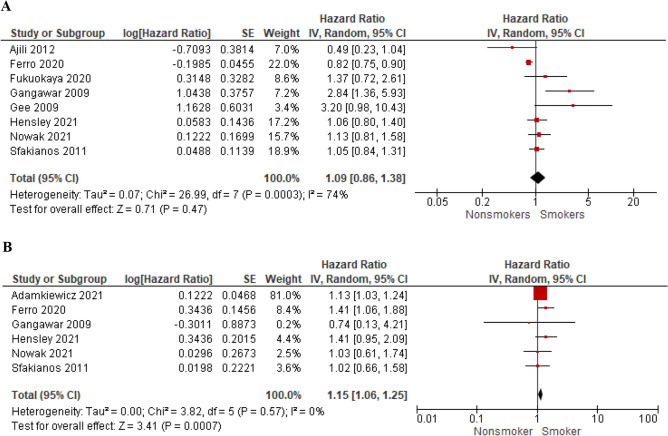
The association of smoking with recurrence-free survival (**A**) and progression-free survival (**B**) in BCG-treated patients with non-muscle invasive bladder cancer

We selected 64 eligible studies, including 28,617 patients with non-muscle invasive bladder cancer and reported oncological outcomes according to the smoking status (Figs. 3, 4, 5, 6, 7, 8, 9). Thirty-one studies were eligible for the analysis of recurrence or progression rates among smokers and non-smokers. Among 8674 patients included in the above analysis, there were 2738 non-smokers (31.6%) and 5936 smokers (68.4%). Seventeen of included studies provided detailed information on the smoking status and 1490 (33.8%) were current, 1477 (33.5%) former and 1438 (32.6%) and never smokers, respectively. The median length of follow-up was 45.8 months (range between quartiles 30–60 months).

**Table 1:** Characteristics of studies included (*N* = 64) for meta-analysis

Study	Smoking categories	Reported outcome	Continent/country	Study type	Years	Pts. no	Mean/median age	Pathological stage	Pathological grade	Adjuvant therapy	Follow-up (months)
Abufaraj et al. (2017)	Never, former, current	HR	EU, USA	R	NR	827	67	Ta 56; T1 41.8; Tis 2.2	G1 23.6; G2 32.3; G3 44.1	BCG 16.3; CHT 2.7	55
Adamkiewicz et al. (2021)	Smoking history (yes/no)	HR	EU	R	NR-2020	125	69	Ta 42.4; T1 57.6	LG 55; HG 45	BCG 100	22–24
Ajili et al. (2012)	Smoker or nonsmoker	HR, raw values	AFR	R	2000–2007	112	63.9	Ta 60.7; T1 39.3	G1 39.2; G2 43.8; G3 17	BCG 100	30
Allard et al. (1995)	Never, former, current	HR, raw values	CA	P	1990–1992	368	65	Ta 78.8; T1 21.2	G1 32.2; G2 53.8; G3 12	BCG 17.4; CHT 2.2	23.7
Blute et al. (2017)	Smoking history (yes/no)	HR	USA	R	2000–2014	727	69.8	Ta 70.7; T1 23.5; Tis 5.8	LG 49; HG 51	BCG 40.1; CHT 8.1	44.4
Cao et al. 2016)	Never, former, current	HR, raw values	AS, EU	R	2008–2013	242	64	Ta 57; T1 43	LG 61.2; HG 38.8	BCG 0; CHT 100	21
Cantiello et al. (2018)	Never, former, current	HR	EU, USA	R	2002–2012	1155	71	T1 100	HG 100	BCG 100	48
Carta et al. (2018)	Never, former, current	Raw values	EU	R	1997–2000	160	NR	NR	LG 25.6; HG 74.4	BCG NR, CHT NR	55.6
Chen et al. (2008)	Nonsmokers, former, quitters, continued smokers	HR, raw values	AS	R	1997–2005	413	67	Ta 58.4; T1 41.6	LG 71; HG 29	BCG 16; CHT 56.9; BCG/CHT 20.6	36
Cheng et al. (2021)	Smoking history (yes/no)	HR	AS	R	2013–2017	314	65	Ta 56; T1 44	LG 33.3; HG 66.7	BCG or CHT 16.2	48
Cheng et al. (1999)	Never, former, current	Raw values	USA	R	1987–1992	83	71	T1 100	LG 34; HG 66	BCG 13.2; CHT 19.7	62.4
D’Andrea et al. (2017)	Nonsmoker, ever-smoker	HR	EU, USA, CA	R	NR	918	66–67	Ta 60.5; T1 39.5	G1 20; G2 36.8; G3 43.2	CHT 4.3; BCG 12.9	62
Abd Elwahab et al. (2021)	Smoker or nonsmoker	Raw values	AFR	P	2013–2020	65	61.5	T1 100	G3 100	BCG 100	60
Favilla et al. (2016)	Never, former, current	HR	EU	P	2008–2014	178	69.3	Ta 77.5; T1 22.5	LG 70.8; HG 29.2	BCG 10.7; CHT 73	53
Zhu et al. (2019)	Smoker or nonsmoker	Raw values	AS	P	2017–2018	95	65	Ta 70; T1 25; Tis 5	LG 39; HG 61	BCG NR, CHT NR	18
Ferro et al. (2020)	Smoking status (current/former), never	HR	EU	R	2002–2012	1172	70.3	T1 100	HG 100	BCG 100	47
Fukuokaya et al. (2020)	Smoking history (yes/no)	HR	AS	R	2002–2018	582	73	Ta 60; T1 36.9; Tis 2.9	G1 6.3; G2 46.5; G3 47.2	BCG 23.4	41.3
Gangawar et al. (2010)	nonsmokers (never), smokers, chewers	HR, raw values	AS	R	2006–2008	135	58	NR	G1 50.4; G2/G3 49.6	BCG 54.8; MMC 10.4	14
Garczyk et al. (2021)	Never, former, current	HR, raw values	EU	R	2008–2014	128	66	Tis 19; Tis/TaT1 81	HG 100	BCG 70	66
Garg et al. (2020)	Never, ever	HR	USA	R	2003–2015	1485	73.5	Ta 67.4; T1 26.3; Tis 5	LG 47.5; HG 52.5	BCG NR, CHT NR	70.8
Gee et al. (2009)	Current smoker, history of smoking	HR	USA	R	1991–2003	43	NR	Ta 9; Tis 84	HG 100	BCG 100	60
Grotenhuis et al. (2014)	Never, ever	HR, raw values	EU	R	NR	1269	64	Ta 68; T1 26; Tis 4	LG 61; HG 38	BCG 20; CHT 31	60
Hensley et al. (2021)	Never, ever	HR	USA	R	2000–2018	518	66–69, NR	Ta 45; T1 47.5; Tis NR	LG 11; HG 89	BCG 100	50
Jobczyk et al.(2020)	Smoker or nonsmoker	HR	EU, USA	R	NR	389	68	Ta 63; Tis 3; T1 34	G1 54; G2 35; G3 11	BCG 29	48
Huang et al.(2021)	Smoking history (yes/no)	HR	AS	R	2011–2015	88	64.5	Ta 72.7; T1 27.3	LG 60.2; HG 39.8	BCG NR, CHT NR	60
Hwang et al.(2011)	Smoker or nonsmoker	HR	AS	R	2000–2010	251	67	Ta 64; T1 36	PUNLMP 5.6; LG 62.5; HG 32	BCG 51; Epirubicin 14	34
Kang et al.(2014)	Smoker (prior/current) or nonsmoker	Raw values	AS, USA	R	1992–2009	135	65	Ta 31.9; T1 68.1	G1 23.7; G2 59.3; G3 17	BCG 100	66.7
Khan et al.(2014)	History of smoking vs never	Raw values	AS	R	2008–2012	64	59.9	NR	HG 100	BCG 100	28.4
Kim et al.(2018)	Smoking status (current/former), never	Raw values	AS	R	1999–2014	64	NR	Tis 100	HG 100	BCG 100	NR
Kimura et al.(2018)	Never, former, current	HR	AS, EU, USA, CA	R	NR	1117	67	Ta 58; T1 40; Tis 2	G1 21; G2 35; G3 44	BCG 39.7; CHT 4	64
Kobayashi et al.(2014)	Never, former, current	HR, raw values	AS	R	1986–2016	190	62.9	Ta 100	LG 100	BCG 37.4; MMC 6.3	101.5
Kufukihara et al.(2021)	Never, former, current	HR	AS	R	1999–2017	1097	NR	Ta 70; T1 30	G1 6; G2 54; G3 40	BCG 41	60
Lacombe et al.(2016)	Never, former, current	HR, raw values	CA	P	1990–1992	189	62.8	Ta 77.2; T1 22.8	LG 31.2; HG 68.8	BCG 100	67.2
Lammers et al. (2011)	Never, former/current	HR	EU	P	1998–2004	718	66.5	Ta 78.7; T1 21.3	G1 42.1; G2 47; G3 10.9	BCG NR; CHT 100	30
Li et al. (2017)	Never, former, current	HR, raw values	AS	R	2007–2015	484	64	Ta 83.5; T1 16.5	G1 18.8; G2 65.3; G3 15.9	CHT 71.7, BCG NR	25
Li et al. (2020)	Smoking (yes/no)	HR	AS	R	2013–2017	115	64.5	Ta 100	PUNLMP 37; LG 37; HG 35	CHT 100	24
Li et al. (2020)	Smoking history (yes/no)	HR	AS	R	2012–2015	206	62	Ta 70.4; Tis 3.9; T1 25.7	PUNLMP 8.2; LG 64.6; HG 27.2	BCG NR, CHT NR	42
Lu et al. (2019)	Never, former, current	HR	AS	R	2012–2016	477	64	Ta 75.3; T1 24.7	G1 67; G2 22; G3 11	BCG NR	NR
Lunney et al. (2019)	Never, former, current	Raw values	USA	R	2010–2016	70	65	Ta 58.6; T1 28.6; Tis 9	LG 44; HG 56	BCG NR	31.7
Mano et al. (2015)	Smoking history (former/current vs never)	HR	AS	R	2003–2010	122	68	Ta 43; T1 57	G1G2 39; G3 61	BCG 50; CHT 25	40
Matulewicz et al. (2021)	Never, former, current	HR	USA, CA	R	2014–2020	723	NR	NR-AUA risk groups	NR	BCG 56.6; CHT 17	23.9
Mbeutcha et al. (2016)	Never, former, current	HR	EU, USA, CA	R	1996–2007	1117	67	Ta 60.3; T1 39.7	G1 20.7; G2 35.6; G3 43.7	BCG 26.9; CHT 4;	64
Nowak et al. (2021)	Never, former, current	HR	EU	R	2001–2019	590	66.9	T1 100	G3 100	BCG 100	40
Mitrakas et al. (2019)	Never, former, current	Raw values	EU	R	NR	80	67.5	Ta 18.8; T1 81.2	HG 100	BCG 100	62.7
Ogihara et al. (2016)	Smoking history (yes/no)	Raw values	AS	R	1995–2012	634	68.5	Ta 68.3; T1 31.7	G1G2 62.7; G3 37.3	BCG 45.6	68.1
Ogihara et al. (2016)	Never, former, current	HR, raw values	AS	R	1995–2013	605	68	Ta 68.3; T1 31.7	LG 60.7; HG 39.3	BCG 47.8	68.8
Osch et al. (2018)	Never, former, current	HR, raw values	EU	R	2006–2011	210	71	Ta 66; T1 33; Tis 1	G1 30; G2 36; G3 34	BCG NR; CHT NR	4.21
Özyalvaçlı et al. (2015)	Smoking (yes/no)	Raw values	AS	R	2008–2013	722	67.5	T1 100	HG 100	BCG 34	24.2
Rink et al. (2012)	Never, former, current	HR	EU, USA, CA	R	1987–2007	2043	67	Ta 61; T1 39	G1 23.6; G2 33.8; G3 42.6	BCG 16.1; CHT 3.8	49
Sawazaki et al. (2021)	Smoking history (yes/no)	HR	AS	R	2014–2018	75	74.7	Ta 38.6; T1 57.3; Tis 4	LG 66.7; HG 33.3	BCG 30.6	37
Serretta et al. (2013)	Never, former/current	HR, event rate	EU	R	2002–2003	395	68	Ta 36.5; T1 63.5	G1 35.9; G2 64.1	CHT 100	48
Sfakianos et al. (2011)	Never, former, current	HR	USA	R	1994–2008	623	76	Ta 35.2; T1 34.5; Tis 30.3	LG 9.6; G3 90.4	BCG 100	80.9
Shen et al. (2016)	Smoking history (never vs former/current)	HR	AS	R	2005–2011	318	65	T1 100	LG 48.8; HG 49.5; unk 1.7	BCG 0; CHT 96.2	53.5
Soria et al. (2018)	Never, former, current	HR	EU, AS, USA	R	1996–2007	1117	68	Ta 58; Tis 2; T1 40	G1 20; G2 36; G3 44	BCG 27; CHT 4	62.7
Shiota et al. (2017)	Never, former/current	HR	AS	R	2010–2013	228	70	Ta 68.9; T1 21.9; Tis 9.2	LG 44.3; HG 55.7	BCG 29.8; CHT 47.8	3.6
Temiz et al. (2021)	Never, former, current	Event rate	AS	R	2015–2018	53	67	Ta 41.5; T1 56.6; Tis 1.9	LG 17; HG 83	BCG 100	11.5
Ucpinar et al. (2019)	Never, former, current	HR, raw values	AS	R	2015–2018	231	64	NR	NR	BGC NR	24
Wang et al. (2020)	Smoking (yes/no)	HR	AS	R	2010–2014	606	70	Ta 61.7; T1 29.4; Tis 8.9	G1 18.6; G2 55; G3 26.4	BCG 19.2; CHT 13	44.5
Wyszynski et al. (2014)	Never, former, current	HR	AFR, EU	R	1994–2001	726	NR	TaT1 94; Tis 6	LG 74; HG 26	BCG NR	72
Yang et al. (2021)	Smoking history (Never vs former/current)	HR	AS	R	2014–2018	235	66	Ta 81.7; T1 18.3	LG 57; HG 43	BCG NR	42
Yonekura et al. (2019)	Never, former, current	HR	AS	R	2011–2015	40	73	Ta/Tis 75; T1 25	LG 65.0; HG 35	BCG 7.5	37.9
Yuruk et al. (2017)	Never, former, current	Raw values	AS	R	2005–2012	212	64.7	Ta 50.3; T1 49.7	LG 59.9; HG 40.1	BCG 3.8; CHT 42.8	32
Zeng et al. (2020)	Smoking (yes/no)	Raw values	AS	R	2017–2019	40	65	Ta 72.5; T1 27.5	PUNLMP 25; LG 40; HG 35	BCG NR; CHT 67.5	12
Zhao et al. (2021)	Smoking history (yes/no)	HR	AS	R	2011–2015	104	63.2	Ta 71.3; T1 28.7	LG 61.7; HG 38.3	BCG NR	43.6

*HR* hazard ratio, *P* prospective study, *R* retrospective study, *NR* not reported, *LG* low-grade, *HG* high-grade, *PUNLMP* papillary urothelial neoplasm of low malignant potential, *BCG* Bacillus Calmette-Guerin, *CHT* intravesical chemotherapy, *MMC* mitomycin, *EU* Europe, *AS* Asia, *AFR* Africa, *USA* Unites States of America, *CA* Canada

The characteristics of included studies and patient cohorts are summarized in Table 1. Only a minor part of the studies focused exactly on the role of smoking on NMIBC prognosis, whereas the remaining studies included smoking in uni- or multivariate analysis as a potential confounding factor, but the smoking status was not the main subject of these studies. Among all 64 included studies, patients’ age, tumour pathologic characteristics and additional intravesical treatment (e.g. BCG) differed.

The conducted analyses (Figs. 3, 4, 5, 6, 7, 8, 9) included a different number of studies which provided data to investigate the association between smoking status (current/former/never smoker or smoker/non-smoker) and recurrence or progression. Necessary details, which explain the selection of studies for consecutive analyses were presented in Table 1.

### Recurrence and progression risk in ever smokers

In a meta-analysis of 28 studies with 7885 patients we found that smokers (current/former) are at higher risk for recurrence (OR = 1.68; 95% CI 1.34–2.09; *I*^2^ = 71%; *P* < 0.0001) compared to never smokers (Fig. 3A). Among 5502 smokers, 2487 individuals (45.2%) developed tumour recurrence compared to 825 from 2383 patients classified as non-smokers (34.6%). Sensitivity analysis including only prospective studies confirmed that the risk of recurrence was higher for smokers compared to non-smokers (OR = 2.18 95% CI 1.57–3.02; *I*^2^ = 19%; *P* < 0.0001, Supp. Figure 1). Analysis of 9 studies including 2672 patients regarding the progression risk did not reveal an increased risk for smokers vs never smokers (progression rate 15.9% vs 13% in smokers and non-smokers, respectively; OR = 1.26; 95% CI 0.94–1.68; *I*^2^ = 11%; *P* = 0.12, Fig. 3B).

### Recurrence risk in current smokers and former smokers

Seventeen studies (*N* = 4405) provided exact data on recurrences in the group of current smokers, former smokers and never smokers, respectively. The comparison between the risk of recurrence was also performed between the respective groups: current smokers vs former smokers and current vs never smokers (Fig. 4A, b). Current smokers were at 1.24 higher risk of recurrence (OR = 1.24; 95% CI 1.02–1.50; *I*^2^ = 28%; *P* = 0.03) compared to former smokers and former smokers had an increased risk compared to never (OR = 1.55; 95% CI 1.12–2.14; *I*^2^ = 68%; *P* = 0.008).

Many studies did not provide comprehensive data on recurrence/ progression rates or numbers in the subgroups of smokers and non-smokers, but provided hazard ratios supplemented with 95% confidence interval and those were used for RFS and PFS analysis. Fifty studies were available for analyses of the pooled hazard ratio for recurrence and/or progression.

### Recurrence-free survival according to smoking status

Twenty-seven studies evaluated the effect of ever smoking on recurrence-free survival and seventeen provided more detailed information on the effect of current and former smoking compared to never smoking on RFS. RFS was worse in ever smokers compared to never smokers (HR = 1.26; 95%CI 1.13–1.39; *I*^2^ = 77%; *P* < 0.0001) (Fig. 5) and in current smokers vs never smokers (HR = 1.27; 95% CI 1.09–1.47; *I*^2^ = 67%; *P* = 0.002) (Fig. 6A). In the analysis of pooled hazard ratio, former smokers did not have an evident inferior RFS compared to those who never smoked (HR = 1.14; 95% CI 0.97–1.34; *I*^2^ = 70%; *P* = 0.10) (Fig. 6B). Sensitivity analysis was also performed and confirmed the higher risk of recurrence for ever smokers compared to never smokers (please see supplementary Fig. 2A to compare with Fig. 5.).

### Progression-free survival according to smoking status

A meta-analysis of 13 studies showed that smokers have worse PFS (HR = 1.18; 95% CI 1.08–1.29; *I*^2^ = 7%; *P* < 0.001) compared to never smokers (Fig. 7). Sensitivity analysis was also performed and confirmed the higher risk of progression for ever smokers compared to never smokers (please see Supplementary Fig. 2B to compare with Fig. 7).

### High-risk subgroup- effect of smoking history on RFS and PFS

Subgroup analysis of RFS in high-risk NMIBC patients (> 80% of the cohort studied in each paper or subgroup analysis available) included six studies and showed that smoking status did not influence RFS (HR = 1.02; 95% CI 0.85–1.22; *I*^2^ = 66%; *P* = 0.85) (Fig. 8A). However, the data retrieved from five studies including the analysis of PFS in patients with high-risk NMIBC demonstrated that ever smokers had compromised PFS compared to never smokers (HR = 1.30; 95% CI 1.09–1.55; *I*^2^ = 0%; *P* = 0.004) (Fig. 8B).

### BCG-treated subgroup- effect of smoking history on RFS and PFS

Subgroup analysis of RFS in BCG-treated patients (100% of the cohort studied in each paper or subgroup analysis available) included eight studies and showed that smoking status did not influence RFS (HR = 1.09; 95% CI 0.86–1.38; *I*^2^ = 74%; *P* = 0.47) (Fig. 9A). However, the data retrieved from six studies including the analysis of PFS in patients receiving BCG demonstrated that ever smokers had compromised PFS compared to never smokers (HR = 1.15; 95% CI 1.06–1.25; *I*^2^ = 0%; *P* < 0.001) (Fig. 9B).

## Discussion

In the current meta-analysis, we sought to determine the effect of smoking on NMIBC recurrence and progression. Our meta-analysis shows that smokers (former/current) have a worse prognosis than non-smokers diagnosed with NMIBC. Both RFS and PFS are compromised in patients who have ever smoked compared to never smokers. A separate analysis of the recurrence rates confirmed an increased risk of recurrence but not progression in smokers compared to non-smokers.

The initial evidence of the smoking impact on bladder cancer prognosis comes from the cohorts treated with neoadjuvant chemotherapy (NAC) and radical cystectomy (RC), in which smoking status increased cancer-specific mortality (CSM) (Cacciamani et al. 2020; Crivelli et al. 2014). A meta-analysis by Cacciamani et al. showed that smoking affects the pathological response to NAC and CSM after NAC and RC. Active smokers had lower NAC response rates and the burden of CSM and OM and bladder cancer recurrences were higher compared to nonsmokers (Crivelli et al. 2014). Our meta-analysis was meant to provide the rationale for generalizing the previous observations on the detrimental effect of smoking on prognosis in locally advanced bladder cancer onto NMIBC.

To date several retrospective analyses presented conflicting results on the effect of smoking on NMIBC prognosis (Grotenhuis et al. 2014; Ogihara et al. 2016; D’Andrea et al. 2017; Ślusarczyk et al. 2019). It has been also recognized that former smokers perform better than those who continue smoking in NMIBC cohorts (Chen et al. 2007; Rink et al. 2013). Importantly, some studies show that smoking cessation might not only prevent recurrence and progression (Chen et al. 2007; Rink et al. 2013) but also impact survival as reported by the recent cross-sectional real-world experience study (Karlsson et al. 2021). In our meta-analysis, a subgroup analysis of 17 papers summarized by pooled hazard ratio demonstrated that current, but not former smoking is associated with unfavourable RFS compared to never smoking, which might suggest that smoking cessation improves the RFS. Moreover, the subgroup analysis of the other 17 papers, providing contingency tables for odds ratio calculation, revealed that current smokers are at increased risk for recurrence compared to former smokers. It has been already reported that smoking cessation more than 15 years before bladder cancer diagnosis reduces recurrences regardless of the intensity or duration of past smoking (Ogihara et al. 2016). On the other hand, a prospective trial by Seretta et al. did not confirm the current smoking as the risk factor for recurrence after TURBT as the observed 8.4% risk reduction in quitters failed to be statistically significant (Serretta et al. 2020). Noteworthy, the cessation time in this study has not exceeded 8.5 months. Thus, since the smoking-free interval might require to be longer to unsheathe the benefit in prognosis, our classification of patients as former smoking as simply individuals that stopped smoking before TURBT, might prevent the results from generalizing to some clinical settings.

Several studies suggest the relationship between smoking and the reduced effectiveness of intravesical adjuvant therapy (Abd Elwahab et al. 2021; Lammers et al. 2011; Rink et al. 2012). BCG immunotherapy was reported to be less effective in smokers compared to non-smokers (Ślusarczyk et al. 2019; Abd Elwahab et al. 2021; Lammers et al. 2011). Another study confirmed that greater lifetime smoke exposure (especially over 20 pack-years) confers a risk factor for recurrence and progression of NMIBC treated with BCG (Andrade et al. 2020 Aug). Such observation might reflect not only the carcinogenesis and mutagenesis in urothelial cells induced by smoke but also the immunomodulatory effects of tobacco smoking. Our meta-analysis of the NMIBC subgroup treated with BCG therapy showed worse PFS but not RFS in smokers compared to non-smokers, which emphasizes the importance of early smoking cessation to avoid progression and spare the bladder. The discrepancy between the clear effect of smoke on PFS despite no effect on RFS might come from high end-point prevalence (recurrence) despite a limited sample size. Noteworthy, in the majority of analyses high-grade and low-grade recurrences are not counted separately despite their completely different prognostic value. Since prospective evidence of the smoking effect on BCG therapy outcomes is lacking, clear recommendations in this area might be yet from clinical implementation. However, based on the results presented and the general harms related to smoking, patients with high-risk NMIBC should be strongly advised to quit smoking when qualified for BCG immunotherapy and informed about the potential increased risk of therapy failure when smoking. Implementation of novel immunotherapies targeting immune checkpoint (IC) raises the question of its applicability in certain groups of patients, including smokers. Current evidence from lung cancer suggests that due to higher mutational burden, smokers might achieve better responses to IC inhibitors than never smokers (Dai et al. 2021).

In summary, tobacco smoking has a detrimental effect on non-muscle invasive bladder cancer management. Smoking cessation should be counselled in each patient due to the general harm it causes and the worsening of bladder cancer prognosis. Bladder cancer was ranked as the most expensive cancer per capita to treat (Cost considerations in the management of bladder cancer [Internet]. 2021). High expenses for bladder cancer management come from several factors, one of which is the recurrent disease requiring repeated surgical procedures (e.g., TURBT) and intensive cystoscopic surveillance and intravesical therapy (Svatek et al. 2014). Smoking surely increases the cost of NMIBC treatment as according to our meta-analysis, the recurrence occurs 68% more commonly in ever smokers compared to never smokers. Current smoking at the time of UCB diagnosis is associated with an additional 24% increase in the recurrence risk compared to a history of smoking in the past. Urological advice and assistance in quitting smoking are suboptimal, despite the fact of their relevance and efficacy (Sosnowski et al. 2016). A prospective trial showed that a short 5 min brief smoking cessation intervention increased the rate of UBC patients who quit smoking (12.1 vs 2.6%) compared to patients under usual care (Bjurlin et al. 2013). Our study demonstrates the detrimental effect of smoking on further tumour recurrence risk and delivers another rationale for cessation intervention by urologists and proposal of nicotine replacement therapy or another quitting programme for highly addicted individuals. Other studies including a cessation group suggest that former smokers (especially those early quitting, 15 years before diagnosis) perform better than those who continue smoking (Ogihara et al. 2016; Chen et al. 2007; Rink et al. 2013). As this meta-analysis did not assess the effect of quitting to smoke after NMIBC diagnosis, no firm conclusions in that clinical setting can be made. Further prospective studies with cessation intervention are required to confirm a worse prognosis in continued smokers compared to those who quitted.

Our meta-analysis revealed several discrepancies that should be evaluated. Although progression-free survival was worse in smokers compared to non-smokers (*P* < 0.001), we failed to validate it in contingency tables evaluating progression risk itself (*P* = 0.12). The discrepancy between the evidenced risk of compromised PFS (HR) but not increased progression risk (OR) might signalize the bias in the selection of the studies. Significant differences in follow-up time and characteristics of cohorts analyzed in included studies might therefore explain the discrepancy. The extraction of the data for this meta-analysis from studies that did not aim to focus on the smoking effect on prognosis as the main research question and, therefore, did not show comprehensive data on smoking, must be considered as another limitation. Analyses of event-free survival and risk of an event do not lead to the same conclusions and, therefore, presented effects of smoking on progression should be interpreted with caution. Nevertheless, the detrimental effect of smoking on PFS was also observed in the sensitivity analysis (supplementary Fig. 2.) and subgroup analyses- in BCG-treated individuals as well as in the high-risk-only NMIBC cohort. Another issue is the lack of adjustment for other prognostic factors (e.g., grade, T category) when calculating recurrence/progression risk (odds ratio), but in the majority of studies providing RFS/PFS assessment, the hazard ratio was adjusted for other confounders. The higher prevalence of aggressive cancer in smokers than in non-smokers (Pietzak and Malkowicz 2014) is a non-negligible confounder, contributing to the potential bias in our analysis. On the other hand, some studies do not clearly confirm the association between baseline tumour characteristics and smoking status. In the study of Barbosa et al. analysing a large cohort of 1859 patients, smokers constituted 82% of low-risk and 82% of high-risk NMIBC (Barbosa et al. 2018). It should be, however, noted that among former smokers, duration and amount of smoking correlated with tumour aggressiveness (Barbosa et al. 2018). Another study by Pietzak et al. suggested that heavy smokers (≥ 30 pack-years) are at increased risk of high-grade tumour and MIBC at presentation compared to light smokers (< 30 pack-years) and non-smokers (Pietzak et al. 2015). Therefore, although higher aggressiveness of tumours in current smokers might result in certain confounding in itself, it seems that in the end what matters most is the smoke load.

Limitations of the above meta-analysis result from the inclusion of studies with moderate- to high-risk of bias (26 and 7 studies, respectively). Almost all included studies were retrospective and the majority of them did not focus directly on smoking status and did not address its prognostic value as a main research question. Consequently, smoking status reporting differed between studies (as shown in Table 1). Possible bias from not reporting the effect of smoking in other published studies, in which no effect of smoking was found, cannot be ruled out. The heterogeneity of studies included in the RFS analysis was moderate to high, which limits generalizing the results to every clinical setting. Noteworthy, a sensitivity analysis with only prospective studies was characterized by low heterogeneity and confirmed that smokers have a higher risk of recurrence than non-smokers (supp. Figure 1). To exclude another potential confounder, we have also performed a subgroup analyses for European/North American and Asian patients regarding RFS and PFS, which further confirmed that the effect of smoking remains significant in patients of different geographic origin (see Supp. Figure 3A–D). We also did not study the effect of smoking intensity and load on prognosis, because of the heterogeneity of reported data in single papers. Andrade et al. showed that smoke load, especially over 20 pack-years influenced recurrence and progression risk in BCG-treated pT1 NMIBC (Andrade et al. 2020). Rink et al. showed that heavy long-term smokers (≥ 20 years; ≥ 20 cigarettes per day) had the worst prognosis followed by light long-term (≥ 20 years; < 20 cigarettes per day), heavy short-term and light long-term smokers (Rink et al. 2013). Although a detailed analysis of smoking load or intensity would be more informative, we attempted to control this bias by providing a subgroup analysis.

## Conclusions

In conclusion, patients with non-muscle invasive bladder cancer and a history of smoking have a worse prognosis regarding recurrence-free and progression-free survival compared to non-smokers. Current smokers have a higher risk of recurrence than former smokers, so smoking cessation should be always counselled and referral for replacement nicotine therapy should be proposed. Smokers treated with intravesical adjuvant BCG have worse progression-free survival when compared to non-smokers. Prospective studies assessing the effect of smoking on NMIBC prognosis are required to confirm our findings.

## Supplementary Information

Below is the link to the electronic supplementary material.

Supplementary file1 (DOCX 176 KB)

## Data Availability

The extracted data can be obtained from the corresponding author upon reasonable request.
