# Supplementary material for: The impact of smoking on recurrence and progression of non-muscle invasive bladder cancer: a systematic review and meta-analysis
Source: J Cancer Res Clin Oncol. 2022 Nov 21;149(6):2673–91. doi: 10.1007/s00432-022-04464-6 (PMC10129946; doi:10.1007/s00432-022-04464-6)
Supplement: Supplementary file 1 — Supplementary file1 (DOCX 176 KB) [file 432_2022_4464_MOESM1_ESM.docx]

**Supp. Fig.1. Sensitivity analysis including only prospective studies to assess the risk of NMIBC recurrence in ever smokers compared to never smokers.**

**
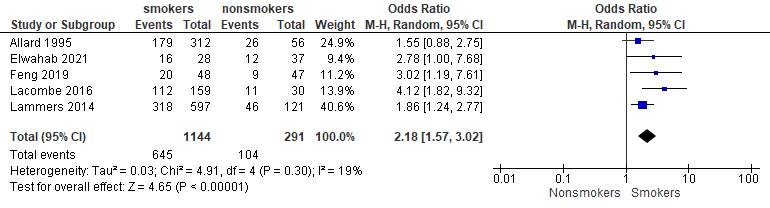
**

**Supp. Fig.2. Sensitivity analysis for the risk of recurrence (A) and progression (B) of NMIBC- comparison between ever smokers and never smokers.**

**A**

**
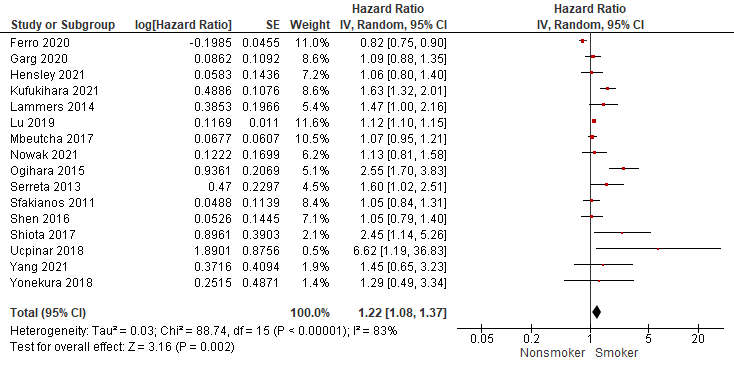
**

**B**


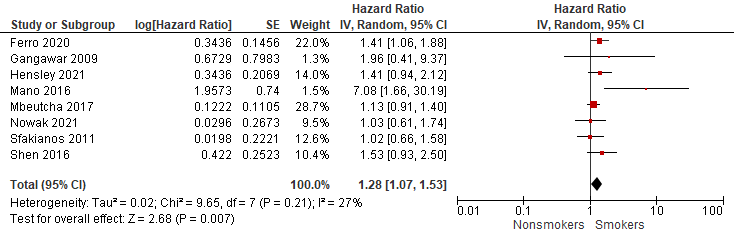


Studies with less reliable reporting on smoking status were excluded.

**Supp. Fig.3. The association of smoking with recurrence-free survival in (A) Asian and (B) European or North American patients respectively. The association of smoking with progression-free survival in (C) Asian and (D) European or North American patients with non-muscle invasive bladder cancer.**

**A**

**
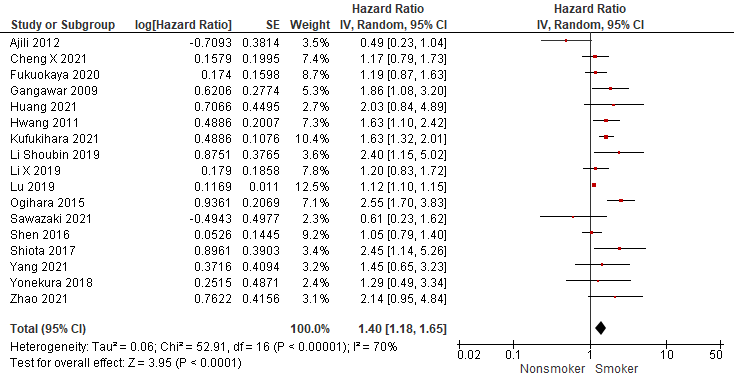
**

**B**

**
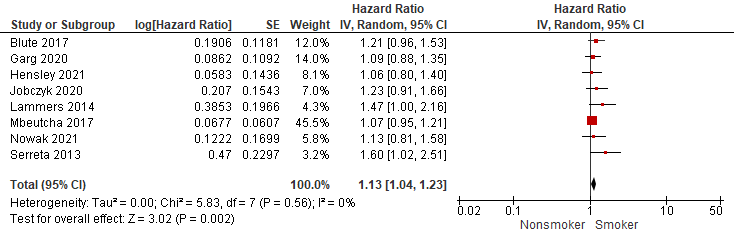
**

**C**

**
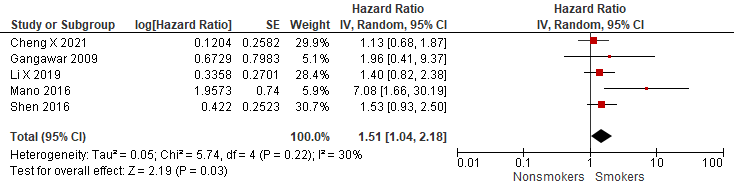
**

**D**

**
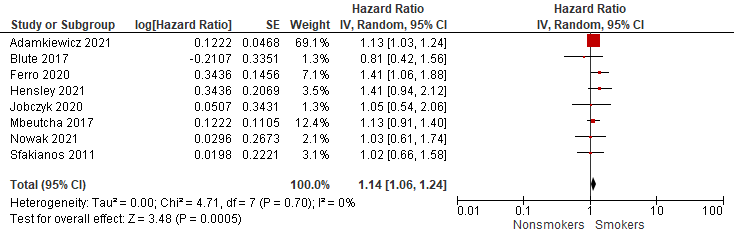
**

**Supp. Fig. 4. Funnel plots of recurrence-free survival in current smokers compared to never smokers (A) and former smokers compared to never smokers (B) in meta-analysis.**

**A
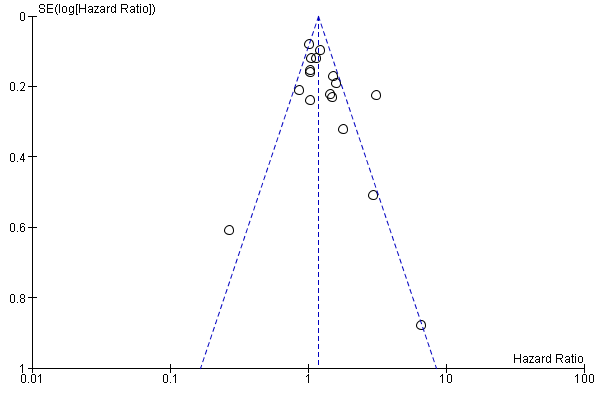
**

**B** **
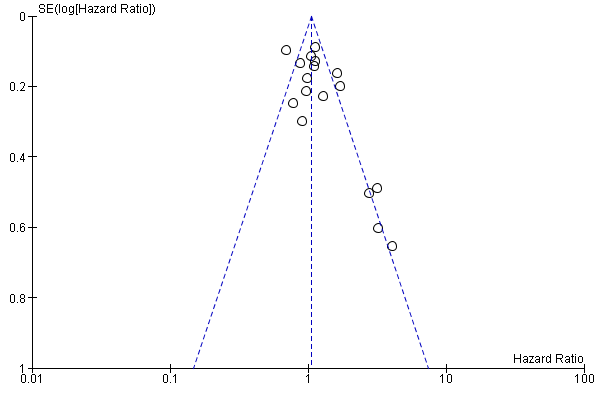
**

**Supp. Fig. 5. Funnel plots of recurrence-free survival (A) and progression-free survival (B) in smokers compared to nonsmokers in meta-analysis.**

**A
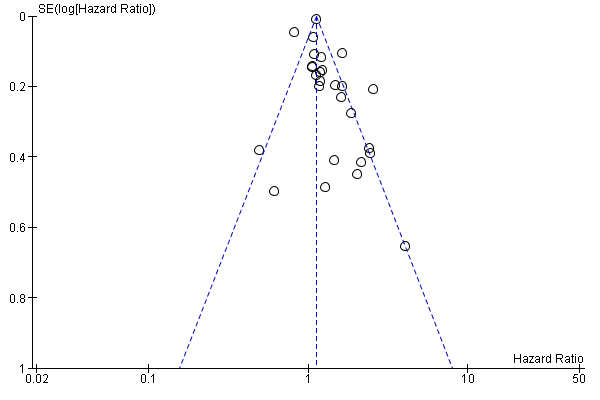
**

**B**

**
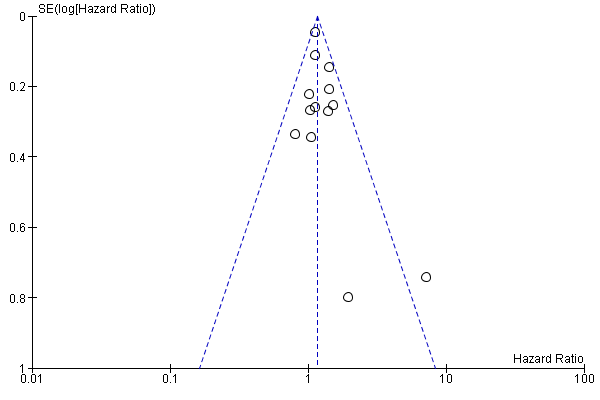
**

**Supp. Fig.6. Funnel plots of recurrence (A) and progression (B) risk in smokers compared to nonsmokers in meta-analysis.**

**A** **
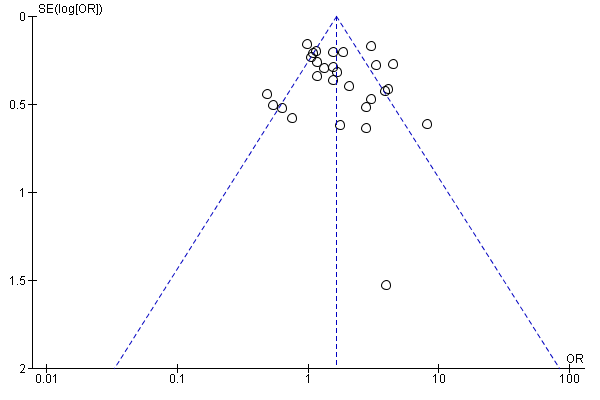
**

**B**
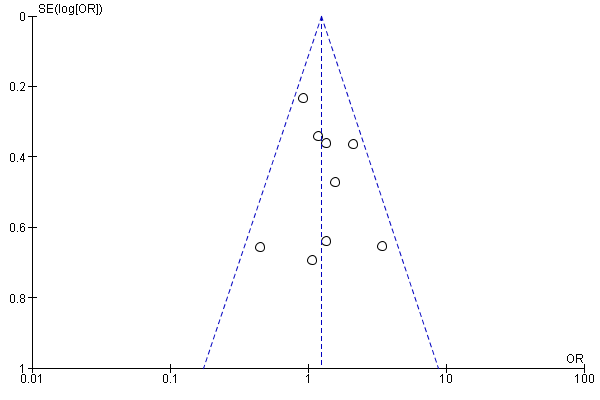


**Supp. Fig.7. Funnel plots of recurrence risk in current compared to former smokers (A) and former compared to never smokers (B) in meta-analysis.**

**A**

**
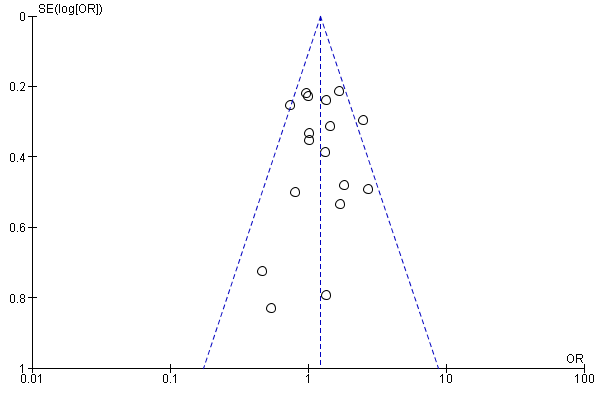
**

**B**

**
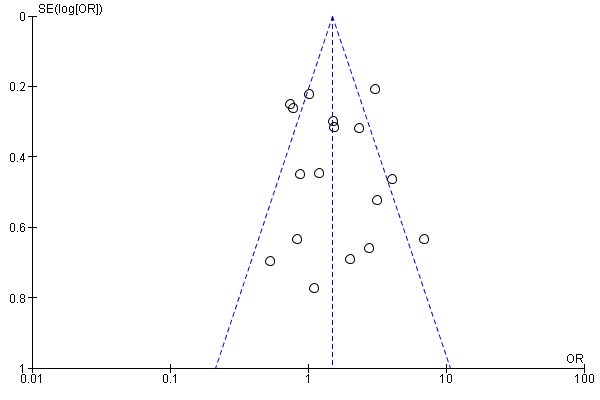
**

**Supp. Fig.8. Funnel plots of recurrence-free survival (A) and progression-free survival (B) in smokers compared to nonsmokers in the subgroup of BCG-treated patients- meta-analysis.**

**A**

**
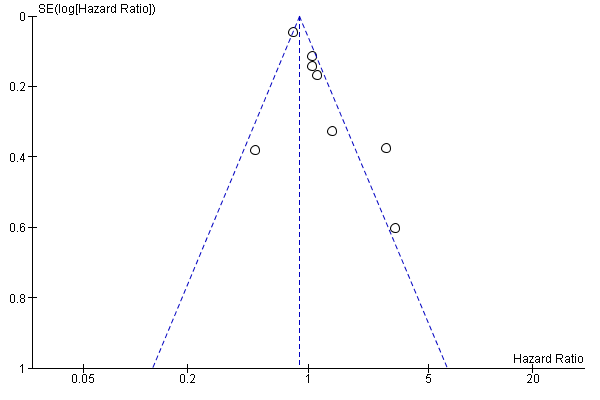
**

**B
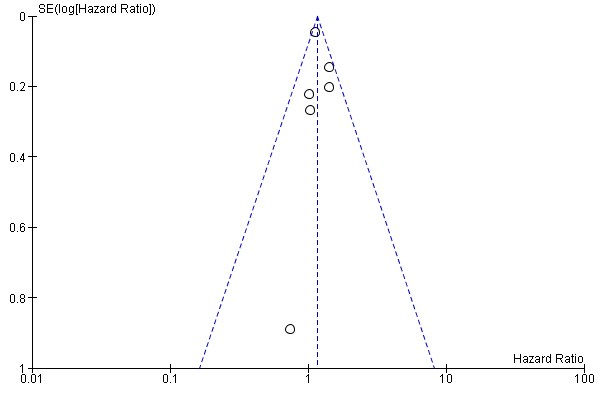
**
